# Size-Related Quality Characteristics of Cage-Reared Butter Catfish (*Ompok bimaculatus*) in a River Basin

**DOI:** 10.3390/ani16040663

**Published:** 2026-02-19

**Authors:** Chatchai Sangpud, Thirarat Kaewchamnong, Sujaree Kaewkong, Manorot Borirak-arawin, Chawanrat Srinounpan, Manat Chaijan, Ari Wibowo, Worawan Panpipat

**Affiliations:** 1 Faculty of Science and Technology, Nakhon Si Thammarat Rajabhat University, Nakhon Si Thammarat 80280, Thailand; chatchai_san@nstru.ac.th (C.S.); thiraratana_kae@nstru.ac.th (T.K.); sujaree_kae@nstru.ac.th (S.K.); manorot_bor@nstru.ac.th (M.B.-a.); chawanrat_sri@nstru.ac.th (C.S.); 2 Food Technology and Innovation Research Center of Excellence, Department of Food Industry, School of Agricultural Technology and Food Industry, Walailak University, Nakhon Si Thammarat 80161, Thailand; cmanat@wu.ac.th; 3 Department of Animal Science, Faculty of Agriculture, Mulawarman University, Pasir Balengkong Rd, Gunung Kelua Campus, Samarinda 75123, East Kalimantan, Indonesia; arikarkun@yahoo.com

**Keywords:** butter catfish, body size, nutritional composition, quality, Geographical Indication (GI)

## Abstract

This study evaluated whether body size could serve as a quality and traceability marker for cage-reared butter catfish (*Ompok bimaculatus*) from the Pak Phanang Basin, Nakhon Si Thammarat, Thailand, in support of Geographical Indication (GI) certification. Fish were classified into three commercial size grades—small (12–15 fish/kg), medium (6–10 fish/kg), and large (3–5 fish/kg)—corresponding to fish harvested after 6, 8, and 12 months of rearing, respectively, with mean body weight and total length of 75 ± 7 g and 19.8 ± 1.1 cm (small), 120 ± 9 g and 25.8 ± 2.1 cm (medium), and 260 ± 10 g and 32.2 ± 2.8 cm (large). Dorsal muscle samples were comparatively analyzed to assess size-related differences in physicochemical properties, nutritional composition, and microbiological quality. Proximate analysis showed that moisture, lipid, ash, and total energy contents increased significantly with fish size (*p* < 0.05), whereas protein and carbohydrate contents did not differ significantly among size groups (*p* > 0.05). Small fish exhibited slightly higher muscle pH (7.02 ± 0.18) than medium and large fish (6.65 ± 0.11 to 6.66 ± 0.25) (*p* < 0.05). Flesh color was characterized by a pale whitish to slightly yellow appearance with high lightness (L*; *p* < 0.05), while redness (a*) and yellowness (b*) did not differ significantly among size groups (*p* > 0.05). Mineral composition varied with size, with large fish containing significantly higher levels of calcium and magnesium (*p* < 0.05). Amino acid profiling identified 17 amino acids, including eight essential amino acids (EAA) and nine non-essential amino acids (NEAA), with EAA being more abundant in large fish—particularly leucine and lysine—indicating improved protein quality, while NEAA were dominated by glutamic and aspartic acids across all size groups. Fatty acid analysis revealed higher proportions of polyunsaturated fatty acids (PUFA) and greater unsaturated fatty acid/saturated fatty acid (UFA/SFA) ratios (1.7–1.8) in medium and large fish. Microbiological assessment showed a decrease in total viable counts with increasing fish size, while *Escherichia coli*, *Staphylococcus aureus*, and *Salmonella* spp. were not detected in any size group, as these microorganisms are commonly used as key hygiene and food-safety indicators in fish products (*E. coli* for fecal/handling hygiene, *S. aureus* for human-handling contamination, and *Salmonella* spp. as a major foodborne pathogen). Overall, body size was associated with consistent variations in physicochemical characteristics, nutritional composition, and microbial quality of Pak Phanang Basin butter catfish. These findings provide baseline compositional and safety markers that can support product specification development and GI documentation.

## Simple Summary

Butter catfish raised in cages in the Pak Phanang Basin (Nakhon Si Thammarat, Thailand) are sold in different market sizes, but it is unclear whether size can help verify product quality and support Geographical Indication (GI) certification. This study compared dorsal meat from small, medium, and large fish to link size with key quality traits. Larger fish had higher moisture, fat, and energy, while protein levels were similar across sizes. Small fish showed slightly higher flesh pH, and all groups had a pale whitish to slightly yellow color. Large fish contained more calcium and magnesium and higher levels of key essential amino acids, especially leucine and lysine, suggesting better protein quality. Medium and large fish also showed a healthier fat profile with more polyunsaturated fats. Total bacterial counts decreased with increasing size, and no harmful bacteria were detected in any group. These findings provide useful baseline markers to help describe, standardize, and document Pak Phanang Basin butter catfish for consumer confidence and GI requirements.

## 1. Introduction

Butter catfish (*Ompok bimaculatus*) is an economically and nutritionally important freshwater species in Southeast Asia, valued for its delicate flesh and high protein content [1]. In Nakhon Si Thammarat Province, particularly within the Pak Phanang Basin, cage-based production in freshwater and semi-open systems has expanded to meet increasing demand while reducing pressure on wild stocks [2]. As with many aquaculture products, market acceptance and safety are influenced by water quality management [3], feed and health management [4], and stocking density [5]. In addition, harvest size is directly linked to commercial grading and consumer preference and can influence the physicochemical properties, nutritional value, and microbiological status of fish products.

Body size is a recognized driver of variation in fish quality attributes, including proximate composition, mineral content, and lipid and protein profiles. Larger fish often accumulate more lipids and energy, while smaller fish may show differences in pH and other physicochemical traits associated with growth stage and post-harvest changes [6]. Other studies report size-related differences in nutrient composition and fatty acid profiles in fish fillets and by-products [7]. Reviews further highlight that size—together with species and habitat—contributes to variation in macro- and micronutrients, including essential minerals and long-chain polyunsaturated fatty acids relevant to human nutrition [8]. For aquaculture systems supplying local and regional markets, defining size-related characteristics is therefore important for product specification, quality control, and value addition.

Beyond quality, traceability and product differentiation are increasingly important for basin-based fishery resources. Geographical Indication (GI) certification is used to protect and promote foods whose quality and reputation are linked to their geographical origin [9,10]. Establishing GI for butter catfish from the Pak Phanang Basin requires systematic baseline evidence to define measurable descriptors that can be verified and communicated as part of a product specification. In this context, compositional information—such as amino acid profiles, fatty acid composition, and mineral content—can function as practical traceability markers when interpreted alongside conventional physicochemical and microbiological indicators, supporting consistent grading and demonstrating product distinctiveness.

Therefore, this study investigated the influence of body size on physicochemical properties, proximate composition, mineral content, amino acid profile, fatty acid composition, color parameters, and microbiological status of cage-reared butter catfish from the Pak Phanang Basin, Nakhon Si Thammarat, Thailand. The findings provide baseline quality and compositional markers to inform product specifications and support GI documentation, while contributing evidence for sustainable aquaculture development in the region.

## 2. Materials and Methods

### 2.1. Fish Samples

Cage culture of butter catfish (*Ompok bimaculatus*) was practiced by Thongchai Farm in Cha-uat District, Nakhon Si Thammarat Province, located within the Pak Phanang River Basin, Thailand. Culture began with stocking fry of 5 cm in length (45 days old) at a density of 150 fish/m^2^. During the first month, fry were reared in fine-mesh cages and fed small-sized commercial pellets (0.2–0.8 mm in diameter; PROFEED, Thai Union Feedmill Public Company Limited, Samut Sakhon, Thailand) containing 40% crude protein; the remaining fraction typically comprises crude lipid (10–15%), carbohydrate (15–25%), ash (<8–10%), crude fiber (1–5%), moisture (<10%), and additives (e.g., vitamins, antioxidants, and preservatives) After one month, fish were transferred to cages with larger mesh sizes and subsequently fed larger pellets (1–1.5 mm; PROFEED, Thai Union Feedmill Public Company Limited, Samut Sakhon, Thailand) three times daily; the diet contained 35–40% crude protein, 5–13% crude lipid, 3–10% crude fiber, 8–12% ash, 20–35% carbohydrates, and 6–10% moisture, with additives such as antioxidants, vitamins, and preservatives. Water in the cages was periodically exchanged to maintain suitable water quality throughout the culture period, as part of routine farm management under commercial operating conditions; however, numerical water-quality parameters (e.g., temperature, dissolved oxygen, pH, and ammonia/nitrite) were not systematically monitored by the research team and were not recorded or provided by the farmer, and therefore could not be reported in this study.

Fish samples representing three commercial size grades were randomly collected: small-sized fish (12–15 fish/kg) after 6 months of rearing, with mean body weight of 75 ± 7 g and total length of 19.8 ± 1.1 cm, medium-sized fish (6–10 fish/kg) after 8 months, with mean body weight of 120 ± 9 g and total length of 25.8 ± 2.1 cm, and large-sized fish (3–5 fish/kg) after 12 months, with mean body weight of 260 ± 10 g and total length of 32.2 ± 2.8 cm. Fish were harvested from three cages per size group (20 fish per cage). Harvesting and killing were conducted on-farm by farmers as part of routine aquaculture practice. Fish were humanely euthanized by immersion in an ice-slurry mixture (ice:water = 5:1) maintained at 0–4 °C until opercular movement ceased, followed by an additional 10 min of immersion to ensure death. The dead fish were packed in ice at a fish-to-ice ratio of 1:3 (*w*/*w*) in polystyrene foam boxes and transported to the laboratory of the Faculty of Science and Technology, Nakhon Si Thammarat Rajabhat University within 1 h.

All samples had a comparable post-mortem period of 3 h prior to analysis. Dorsal muscle samples were excised using a sterile stainless-steel knife, with tissue collected from the middle portion of the body, extending from just behind the head to before the caudal peduncle, to obtain a representative dorsal fillet. The samples were rinsed with distilled water, drained, and gently blotted dry with clean tissue paper, then homogenized to form a composite sample prior to analysis. When immediate analysis was not possible, samples were packed in airtight polyethylene bags and stored at −80 °C for no longer than 1 month before further analysis.

### 2.2. Proximate Composition Analysis and Total Energy Determination

The proximate composition, including moisture, crude protein, lipid, ash, and carbohydrate contents, was determined according to the official methods of the Association of Official Analytical Chemists (AOAC) [11]. Total energy content (cal/g) of the fish muscle was determined using a bomb calorimeter (Model AC600, Leco, St. Joseph, MI, USA) following the standard AOAC procedure [11].

### 2.3. pH Measurement

The pH of fish muscle samples was measured using a calibrated pH meter (SevenCompact S210, Mettler Toledo, Columbus, OH, USA).

### 2.4. Color Measurement

Surface color of the fish fillet was measured using a colorimeter (UltraScan, HunterLab, Reston, VA, USA) based on the CIE L*, a*, b* color system. The parameters recorded were lightness (L*), redness–greenness (a*), and yellowness–blueness (b*) [12].

### 2.5. Mineral Composition Analysis

Major and trace mineral contents in fish muscle, including calcium (Ca), iron (Fe), zinc (Zn), manganese (Mn), sodium (Na), potassium (K), and magnesium (Mg), were determined following AOAC procedures [11] using flame atomic absorption spectroscopy (FAAS). Analysis was conducted using an atomic absorption spectrophotometer (Model AA-6880, Shimadzu, Kyoto, Japan).

### 2.6. Amino Acid Analysis

Amino acid composition of fish muscle was determined according to AOAC methods [11] using an automatic amino acid analyzer (L-8900, Hitachi, Tokyo, Japan).

### 2.7. Fatty Acid Analysis

Fatty acid profiles were analyzed according to BS EN 14103 [13]. Lipids were converted to fatty acid methyl esters (FAMEs) prior to analysis. Quantification was performed using a gas chromatograph equipped with a flame ionization detector (GC-FID; Agilent, Santa Clara, CA, USA).

### 2.8. Microbiological Quality Analysis

Microbiological analyses of fresh fish samples were conducted in accordance with the Bacteriological Analytical Manual (BAM) of the U.S. Food and Drug Administration (FDA). Total plate count (TPC) was determined following BAM Chapter 3 [14]. *Salmonella* spp. were analyzed according to BAM Chapter 5 [15], *Staphylococcus aureus* according to BAM Chapter 12 [16], and coliforms and *Escherichia coli* were enumerated following BAM Chapter 4 [17].

### 2.9. Statistical Analysis

The experiment was conducted using a completely randomized design (CRD) with three independent replications for each size group. All analytical measurements were performed in triplicate. Data were expressed as mean ± standard deviation (SD) from triplicate determinations. Data were subjected to one-way analysis of variance (ANOVA) to evaluate the effects of fish size on the measured parameters. When significant differences were detected, mean comparisons were performed using Duncan’s New Multiple Range Test (DMRT) at a 95% confidence level (*p* < 0.05). Statistical analyses were carried out using IBM SPSS Statistics software (version 21.0). In addition, Pearson’s correlation analysis was performed to evaluate relationships between fish body weight (g) and key nutritional variables (fat, Ca, Mg, Fe, DHA, leucine, and lysine). Simple linear regression was also applied (y = a + bx) to visualize size-related trends, and the coefficient of determination (R^2^) was reported. Correlation and regression results were considered statistically significant at *p* < 0.05, and were presented as heatmap/plots to support interpretation of size-dependent patterns.

## 3. Results

### 3.1. Proximate Composition, Total Energy, and pH

The proximate composition analysis of fresh butter catfish revealed that fish size significantly influenced moisture, fat, and ash contents, while protein and carbohydrate contents were not significantly affected (Table 1). Moisture content differed significantly among size groups (*p* < 0.05), ranging from 68.33 ± 0.04 g/100 g in large fish to 72.66 ± 0.40 g/100 g in small fish. Protein content showed no significant variation among body sizes, remaining within a narrow range of 15.28–16.60 g/100 g (*p* > 0.05). Similarly, carbohydrate content did not differ significantly among size groups (*p* > 0.05), indicating that carbohydrate fractions of the muscle were not markedly influenced by fish size. Fat content was significantly affected by fish size (*p* < 0.05), with large fish exhibiting the highest lipid content (13.35 ± 0.20 g/100 g), followed by medium-sized fish (10.02 ± 0.64 g/100 g) and small fish (8.78 ± 0.03 g/100 g). The ash content was significantly higher in medium- and large-sized fish than in small fish (*p* < 0.05), indicating a higher mineral content in larger fish.

Total energy content varied significantly among size groups (*p* < 0.05) and closely followed the trend observed for fat content. Large fish exhibited the highest energy value (2203 ± 20 cal/g), while medium and small fish showed progressively lower energy contents (1856 ± 84 and 1695 ± 5 cal/g, respectively).

Muscle pH values ranged from 6.65 to 7.02 and were significantly influenced by fish size (*p* < 0.05). Small fish exhibited a slightly higher pH (7.02 ± 0.18) compared with large and medium fish (6.66 ± 0.25 and 6.65 ± 0.11, respectively).

### 3.2. Color

The analysis of flesh color of fresh butter catfish reared in cages showed that fish size significantly affected lightness (L*) (*p* < 0.05) (Table 1). Medium-sized fish exhibited the highest L* value (60.17 ± 4.42), which was significantly higher than that of small fish (57.42 ± 4.98). The largest fish showed an intermediate L* value (48.91 ± 2.10) and did not differ significantly from either medium- or small-sized fish, indicating comparatively darker flesh.

For the a* (red–green) and b* (yellow–blue) color parameters (Table 1), no significant differences were observed among size groups (*p* > 0.05). The a* values ranged from –2.98 to –2.41, while b* values ranged from 6.88 to 9.23, indicating that flesh color remained relatively consistent across all sizes.

### 3.3. Mineral Composition

The mineral composition of butter catfish muscle varied according to fish size, with significant differences observed for Ca, Mg, and Fe (*p* < 0.05), while other minerals remained relatively stable across size groups (Table 2). Large fish exhibited the highest concentrations of Ca (1455 ± 78 mg/100 g) and Mg (1310 ± 50 mg/100 g), whereas the small fish showed significantly lower levels (1043 ± 22 mg/100 g and 1114 ± 4 mg/100 g, respectively). For Fe, large fish contained 18 ± 3 mg/100 g, medium fish contained 12 ± 3 mg/100 g, and small fish contained 7 ± 1 mg/100 g. For Zn, Mn, Na, and K, no significant differences were observed among size groups (*p* > 0.05).

### 3.4. Amino Acid Composition

The analysis of amino acid composition in the flesh of cage-reared butter catfish of different body sizes identified a total of 17 amino acids, comprising both essential amino acids (EAA) and non-essential amino acids (NEAA) (Table 3). Overall, the amino acid profile was dominated by NEAA, particularly glutamic acid and aspartic acid, which were consistently the most abundant amino acids across all size groups. Glutamic acid ranged from 69.85 to 86.59 mg/g, followed by aspartic acid (48.74–56.81 mg/g), confirming their predominant contribution to muscle protein composition.

Fish size significantly influenced the concentration of several amino acids (*p* < 0.05). Large fish generally exhibited higher levels of many amino acids, particularly glutamic acid, aspartic acid, leucine, lysine, and methionine, compared with medium and small fish. Among the eight essential amino acids detected—histidine, isoleucine, leucine, lysine, methionine, phenylalanine, threonine, and valine—distinct size-dependent trends were observed. In contrast, histidine, threonine, and phenylalanine tended to increase in smaller fish. Larger fish, which contained significantly higher levels of glutamic acid. In addition, glycine, alanine, and serine were present at appreciable levels, particularly glycine and alanine in large and small fish. Amino acids present in lower concentrations included cysteine and proline. The relatively higher proline content was observed in smaller fish (*p* < 0.05). Tryptophan and cystine were not detected in any size group.

### 3.5. Fatty Acid Composition

The fatty acid composition of cage-reared butter catfish exhibited clear size-dependent variation in the relative distribution of saturated fatty acids (SFA), monounsaturated fatty acids (MUFA), and polyunsaturated fatty acids (PUFA) (Table 4).

Long-chain fatty acids (LCFA) constituted a major proportion of total fatty acids across all size groups, with the highest contribution observed in small fish (35.01%), followed by medium (29.99%) and large fish (27.89%). Palmitic acid (C16:0) and stearic acid (C18:0) were the dominant saturated fatty acids. MUFA represented a substantial fraction of the lipid profile in butter catfish muscle, accounting for approximately 30–32% of total fatty acids across all size groups. Oleic acid (C18:1 n-9) was the predominant MUFA (26.10–27.63%), followed by palmitoleic acid (C16:1 n-7) and eicosenoic acid (C20:1 n-9). PUFA showed more pronounced size-related variation, contributing approximately 20.75% and 20.33% of total fatty acids in medium and large fish, respectively, but only 6.97% in small fish.

Omega-3 (n-3) PUFA were present at comparatively lower levels. Eicosapentaenoic acid (EPA; C20:5 n-3) was detected in medium and large fish (0.42–0.43%) but was not detected in small fish, while docosahexaenoic acid (DHA; C22:6 n-3) exhibited a clear size-related decline (2.20% in medium fish, 2.00% in large fish, and 0.28% in small fish). The unsaturated-to-saturated fatty acid (UFA/SFA) ratio further highlights size-dependent differences in lipid quality, ranging from 1.1 in small fish to 1.7–1.8 in medium and large fish.

### 3.6. Relationship Between Body Size and Key Nutritional Components

**Figure 1:**
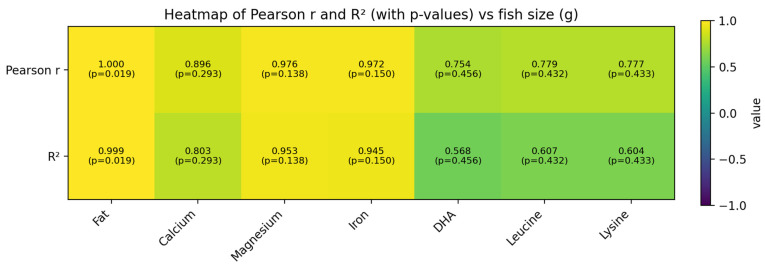
Heatmap of Pearson correlation (r), coefficient of determination (R^2^), and *p*-values between fish size and key nutritional variables. Fish size was expressed as body weight (g) for three size groups (75, 120, and 260 g). The heatmap reports Pearson correlation coefficients (r) describing the direction and strength of association between fish size and each variable, and R^2^ values obtained from simple linear regression models (variable = a + b × fish size), indicating the proportion of variance explained by fish size. The corresponding *p*-values (p) for the relationship between fish size and each variable are shown within each cell. Variables included fat, calcium, magnesium, iron, DHA, leucine, and lysine. Note: Because the analysis is based on three size groups (*n* = 3), the statistical results should be interpreted as indicative size-related trends rather than definitive predictive models.

Correlation and linear regression analyses based on fish body weight (75, 120, and 260 g) showed clear size-related trends in several key nutritional parameters (Figure 1). Fat content increased strongly with fish size (r = 1.000, R^2^ = 0.999, *p* = 0.019). Minerals also tended to increase with body size, with positive associations observed for Ca (r = 0.896, R^2^ = 0.803), Mg (r = 0.976, R^2^ = 0.953), and Fe (r = 0.972, R^2^ = 0.945). DHA also showed a positive, moderate relationship with fish size (r = 0.754, R^2^ = 0.568). The key amino acids leucine and lysine showed positive, moderate relationships with fish size (r = 0.779, R^2^ = 0.607 for leucine; r = 0.777, R^2^ = 0.604 for lysine).

### 3.7. Microbiological Quality

The microbiological quality of butter catfish flesh of different sizes reared in cages was evaluated. Total viable count (TVC) tended to decrease with increasing fish size. The smallest fish (3–5 fish/kg) had the highest TVC (1.5 × 10^4^ CFU/g), followed by medium-sized fish (6–10 fish/kg; 1.0 × 10^3^ CFU/g), while the largest fish (12–15 fish/kg) exhibited the lowest TVC (1.3 × 10^3^ CFU/g) (Table 5).

Total coliform counts were highest in small fish (11 MPN/100 g), followed by medium-sized fish (7.2 MPN/100 g), while large fish showed coliform levels below the detection limit (<3 MPN/100 g). *Escherichia coli*, *Staphylococcus aureus*, and *Salmonella* spp. were not detected in any samples, confirming the microbiological safety of cage-reared butter catfish. 

## 4. Discussion

### 4.1. Proximate Composition, Total Energy, and pH

Proximate composition of fresh butter catfish varied with fish size: moisture, fat, and ash differed significantly among size groups, whereas protein and carbohydrate contents showed no significant size-related changes. The observed increase in moisture content in smaller fish is consistent with the commonly reported inverse relationship between lipid and water content in fish muscle [19]. Muscle protein deposition in butter catfish is relatively stable across size classes, as reported previously for several cultured fish species [7,8]. The increase in lipid content with body size reflects lipid accumulation during growth and agrees with previous studies reporting higher fat deposition in larger and older fish [7]. In general, variations in moisture, protein, lipid, and ash contents within the same species may be associated with differences in body size, sexual maturity, and feeding regime [19,20].

The increase in energy content with fish size is primarily attributed to lipid accumulation, as lipids represent the most energy-dense macronutrient in fish muscle [6,21]. These results indicate that larger butter catfish store more energy to support growth and physiological functions.

Variations in pH among size groups may be related to differences in post-mortem metabolism and muscle energy reserves, which are influenced by body size and stress prior to harvest [22]. Pre-slaughter stress and glycogen depletion are known to affect muscle biochemical pathways and the extent of post-mortem pH decline in fish [23]. Immediately after harvest, the pH of fresh fish muscle is typically close to neutral (approximately pH 7.0), reflecting minimal post-mortem biochemical changes and good nutritional quality. As post-mortem glycolysis proceeds, pH generally declines due to lactic acid formation. Fresh fish commonly exhibit pH values in the range of 6.0–6.5, characterized by firm texture and the absence of off-odors, while moderately fresh fish may show pH values between 6.5 and 6.8, with slightly softer flesh but still acceptable quality. In contrast, spoiled fish often present pH values exceeding 6.8, sometimes reaching >7.5, accompanied by a slimy texture and strong ammonia-like odors resulting from protein degradation [24]. Based on this pH progression, the slightly higher pH observed in small fish (>7.0) may indicate a faster rate of post-mortem biochemical change, rather than spoilage per se, as all samples were analyzed under controlled conditions shortly after harvest. Fish size has been reported to significantly influence the rate of degradation, with smaller fish exhibiting a higher surface area-to-volume ratio, making them more susceptible to rapid biochemical and microbial activity due to increased exposure to the surrounding environment. In contrast, larger fish generally undergo slower degradation rates, as their lower surface area-to-volume ratio can reduce heat loss and metabolic acceleration, particularly under cooler conditions [22]. Nevertheless, the pH values observed in all size groups remained within the acceptable range for fresh fish muscle, indicating good overall quality across all samples.

Overall, these findings demonstrate that fish size significantly affects moisture, fat, ash, total energy, and pH, while protein and carbohydrate contents remain relatively constant in cage-reared butter catfish. This size-dependent variation in muscle composition and energy content is consistent with previous reports and provides useful information for nutritional evaluation and feed management in aquaculture systems [7,8].

### 4.2. Color

Flesh lightness varied with fish size: medium-sized fish had the lightest flesh, small fish were darker than medium fish, and large fish showed an intermediate lightness with comparatively darker flesh overall. Variations in lightness may be associated with size-related differences in muscle structure and lipid deposition, which influence light scattering properties of the flesh. Larger fish, which exhibited higher lipid content, may show reduced lightness due to increased light absorption and altered muscle matrix characteristics, resulting in a darker appearance. Conversely, the brighter flesh observed in medium-sized fish may reflect a more compact muscle structure with moderate lipid levels, contributing to higher light reflectance [25,26].

The red–green (a*) and yellow–blue (b*) color parameters were consistent across all size groups, indicating that flesh hue did not vary noticeably with fish size. Overall, the values reflect a pale whitish to slightly yellow coloration typical of freshwater catfish, consistent with previous reports [7,27]. These results indicate that fish size influenced only the lightness of butter catfish flesh, with medium-sized fish exhibiting the highest brightness, while redness–greenness and yellowness–blueness were not significantly affected. Such size-related differences in flesh lightness may have implications for sensory perception, consumer preference, and market value, particularly where visual appearance is an important quality attribute.

### 4.3. Mineral Composition

Mineral composition differed with fish size, mainly for Ca, Mg, and Fe. Large fish had the highest Ca and Mg levels, while small fish had the lowest; Fe also increased with size, being highest in large fish, intermediate in medium fish, and lowest in small fish, whereas other minerals remained relatively stable across size groups. This pattern suggests that mineral accumulation in muscle tissue is influenced by growth-related physiological processes.

The higher Ca and Mg contents in larger fish may be attributed to prolonged mineral deposition during growth, higher cumulative feed intake, and more developed skeletal and muscular systems. Ca and Mg play crucial roles in muscle contraction, enzymatic activity, and energy metabolism, and their accumulation has been shown to increase with fish size and age in several freshwater species [28,29]. Additionally, larger fish generally have lower relative growth rates but higher absolute nutrient retention, which may favor the deposition of structural minerals in muscle tissue. Similar size-dependent increases in Ca and Mg have been reported in cultured catfish and carp, where mineral retention efficiency improved with body weight and feeding duration [29]. Larger fish exhibited higher Fe content, which may be associated with greater accumulation of hemoproteins in the muscle [28,29,30,31].

Zn, Mn, Na, and K were within the typical ranges reported for freshwater fish [7], suggesting that their accumulation may not be determined solely by fish size or age, but is also influenced by dietary composition and water quality in the culture system [30]. The uniform concentrations of Zn, Mn, Na, and K across all size classes highlight the importance of mineral homeostasis in fish muscle tissue. Fish possess tightly regulated physiological mechanisms that maintain essential mineral levels within narrow ranges to support vital metabolic processes, regardless of growth stage or body size [30]. Trace elements such as Zn and Mn are integral to metalloenzymes and antioxidant defense systems, and their tissue concentrations are therefore strictly controlled to prevent deficiency or toxicity [29,32]. Even as fish grow and total mineral intake increases, excess trace minerals may be redistributed to other tissues, stored in non-muscle compartments, or excreted, resulting in relatively stable muscle concentrations [28].

Similarly, Na and K are key electrolytes involved in osmoregulation, nerve impulse transmission, and muscle excitability. Their levels in muscle tissue are primarily regulated by active ion transport across gill and epithelial membranes rather than by fish size or dietary intake alone [33]. In freshwater species, continuous ionic regulation is required to counteract diffusive losses to the surrounding environment, leading to consistent Na and K concentrations under stable rearing conditions [30]. The lack of size-related variation in these minerals observed in the present study suggests effective physiological regulation and a uniform culture environment across all size groups [34].

In contrast, the size-dependent variation observed for Ca and Mg indicates that macro-minerals associated with structural development and muscle function may be less strictly buffered by homeostatic mechanisms. These minerals are required in relatively large quantities for skeletal formation, muscle contraction, and energy metabolism, and their deposition may increase progressively with body size and cumulative nutrient intake [29]. Overall, the results demonstrate that fish size selectively influences the accumulation of certain macro-minerals, while other essential minerals are maintained at relatively constant levels through homeostatic regulation.

From a nutritional perspective, larger butter catfish may provide enhanced dietary benefits related to bone health and neuromuscular function due to their higher Ca and Mg contents, and may also serve as a better source of dietary heme iron because of their higher Fe content. Meanwhile, the stable levels of Zn, Mn, Na, and K across all size groups suggest that butter catfish, irrespective of size, can serve as a consistent and reliable source of essential minerals for human consumption. In this study, phosphorus (P) was not analyzed because it is not routinely quantified by FAAS under our laboratory workflow and is typically determined using alternative methods (e.g., UV–Vis colorimetry/molybdenum blue or ICP-based techniques); therefore, P will be included in future work to provide a more complete mineral profile.

### 4.4. Amino Acid Composition

A total of 17 amino acids (EAA and NEAA) were identified in the flesh of cage-reared butter catfish across size groups. The profile was dominated by NEAA, with glutamic acid and aspartic acid consistently the most abundant, indicating their major contribution to muscle protein composition. This pattern is in agreement with previous reports for freshwater fish species, where glutamic and aspartic acids are the major amino acids and are closely associated with the characteristic umami taste of fish flesh [35,36,37].

The higher abundance of glutamic acid, aspartic acid, leucine, lysine, and methionine in larger fish may reflect enhanced protein accretion, more developed muscle fibers, and greater cumulative nutrient intake during growth. Similar size-related increases in amino acid deposition have been reported in other cultured freshwater species and are often attributed to improved protein retention efficiency as fish mature [35,36].

Leucine and lysine, which play central roles in muscle protein synthesis and metabolic regulation, were significantly higher in large fish than in medium and small fish. Leucine, a branched-chain amino acid, is a key activator of the mammalian target of rapamycin (mTOR) signaling pathway, thereby stimulating muscle protein synthesis and promoting muscle growth, while lysine is often the first limiting essential amino acid in fish-based and plant-based aquaculture diets and is critical for efficient protein utilization, tissue development, and growth performance Adequate lysine availability has been closely associated with improved protein retention and muscle accretion, whereas deficiencies can impair growth and overall health [36,38,39,40]. Consequently, the elevated levels of these amino acids in larger fish reflect more efficient amino acid deposition and a more balanced essential amino acid profile, indicating superior protein quality, higher nutritional value, and greater nutritional completeness of their muscle tissue for human consumption.

The increasing trend in histidine, threonine, and phenylalanine contents in smaller fish suggests that some essential amino acids may be preferentially retained or utilized differently during early growth stages. This variation reflects the dynamic nature of amino acid metabolism, where requirements and deposition patterns change with growth rate, physiological status, and muscle development [37]. Nevertheless, all size groups contained essential amino acids in proportions that meet or exceed FAO/WHO recommended patterns for high-quality protein foods [38], confirming butter catfish as a nutritionally valuable protein source regardless of size.

From a sensory perspective, the predominance of glutamic acid and aspartic acid—both widely recognized as umami-related amino acids—suggests that butter catfish flesh possesses pronounced savory characteristics. Glycine, alanine, and serine—amino acids linked to sweet taste perception—were also detected at notable levels, with glycine and alanine being especially abundant in large and small fish. These sweet-tasting amino acids can contribute to overall flavor balance by softening and complementing umami sensations [35,36]. Notably, glutamic acid, aspartic acid, glycine, and alanine are commonly classified as umami-taste–active amino acids, as they play a synergistic role in enhancing umami intensity and overall palatability in fish and seafood products [41,42,43].

Cysteine and proline occurred at comparatively low levels, although both are important for collagen structure and connective tissue integrity, which can influence flesh texture and elasticity [37]. The relatively higher proline content in smaller fish may reflect a greater proportion of collagen in younger muscle, potentially contributing to a firmer texture. In contrast, tryptophan and cystine were not detected in any size group, likely because they are unstable under acid hydrolysis and during analysis, or may degrade postmortem—an issue commonly reported in amino acid profiling of freshwater fish [35].

Overall, the results demonstrate that fish size influences not only the nutritional quality but also the potential sensory attributes of butter catfish flesh. Larger fish exhibited higher total amino acid and essential amino acid contents, particularly those linked to muscle growth and umami flavor, indicating superior protein quality and flavor intensity. These findings support the selection of larger butter catfish as premium raw material for high-quality fish products and provide valuable scientific evidence for the development of quality standards and potential GI products based on butter catfish.

### 4.5. Fatty Acid Composition

The fatty acid profile of cage-reared butter catfish varied with body size, showing clear shifts in the relative proportions of SFA, MUFA, and PUFA. Rather than reflecting differences in total lipid content, the observed variation among size groups was primarily associated with qualitative shifts in fatty acid profiles, indicating that growth stage influences lipid metabolism and fatty acid allocation in muscle tissue. Palmitic acid and stearic acid were the dominant saturated fatty acids, consistent with fatty acid profiles typically reported for freshwater fish. The prevalence of these SFAs reflects their structural role in muscle membranes and their involvement in maintaining lipid stability under freshwater environmental conditions [21,44].

The relative stability of MUFA proportions among size classes suggests a conserved metabolic role, while the slightly higher MUFA levels in medium and large fish indicate progressive modulation of lipid metabolism with growth rather than changes in lipid quantity. The dominance of oleic acid is characteristic of freshwater fish and reflects its importance in energy metabolism, membrane fluidity, and lipid homeostasis [44,45]. MUFA have also been reported to function as adaptive biochemical markers of feeding ecology and environmental conditions in freshwater species [46,47].

This marked reduction in PUFA proportion in smaller fish suggests limited accumulation of essential fatty acids during early growth stages, possibly due to developmental constraints in desaturase and elongase activity or reduced dietary availability [21,48]. Among omega-6 (n-6) PUFAs, linoleic acid (C18:2 n-6) was predominant (5.93–15.45%), followed by dihomo-γ-linolenic acid (C20:3 n-6) and eicosadienoic acid (C20:2 n-6). This fatty acid pattern is characteristic of freshwater fish and reflects a predominance of plant-based dietary inputs together with relatively high Δ6-desaturase activity, which enables de novo biosynthesis and elongation of C18 precursors into longer-chain n-6 PUFA [47].

The observed size-dependent variation in PUFA composition may therefore be associated with differences in endogenous fatty acid metabolism. Freshwater species are known to possess higher Δ6-desaturase and elongase capacity than marine fish, allowing efficient conversion of linoleic and α-linolenic acids into longer-chain PUFA [21]. The higher proportions of linoleic acid and its downstream metabolites in medium and large fish are consistent with enhanced biosynthetic conversion during later growth stages, whereas the markedly lower PUFA levels in small fish may reflect limited enzymatic capacity or metabolic prioritization toward somatic growth rather than PUFA accumulation. Although desaturase activity was not directly measured in the present study, the fatty acid profiles observed align with established metabolic patterns reported for tropical freshwater species [48,49].

The concentrations of omega-3 PUFA are comparable to those reported for other tropical freshwater species, such as silver barb (*Barbonymus gonionotus*) and Nile tilapia (*Oreochromis niloticus*), which typically contain less than 1% of total fatty acids as EPA and DHA combined [46,50]. Such findings are consistent with the broader understanding that freshwater fish generally exhibit lower levels of long-chain n-3 PUFA than marine species, primarily due to limited dietary availability of these fatty acids and lower overall desaturase flux toward n-3 pathways in freshwater environments. Despite these relatively modest EPA and DHA levels, the overall PUFA profile of Pak Phanang butter catfish remains nutritionally relevant, as freshwater fish primarily rely on endogenous synthesis of long-chain PUFA from shorter-chain precursors rather than direct dietary intake [21,49].

The UFA/SFA ratio also reflected size-related differences in lipid quality, increasing from 1.1 in small fish to about 1.7–1.8 in medium and large fish. These values indicate favorable lipid quality for human consumption and reflect qualitative differences in fatty acid composition rather than total lipid content [51]. Higher UFA/SFA ratios are associated with improved nutritional value and beneficial health effects, particularly through the roles of unsaturated fatty acids in cardiovascular health, neural development, and anti-inflammatory responses [52]. Similar fatty acid quality patterns have been reported in common carp (*Cyprinus carpio*) and rohu (*Labeo rohita*), where favorable lipid indices were linked to feeding ecology and habitat productivity [53,54].

Overall, the distinct size-dependent fatty acid signatures observed in cage-reared butter catfish from the Pak Phanang River Basin reflect the combined influence of growth stage, metabolic regulation, and environmental feeding conditions. These biochemical characteristics may serve as useful markers for production area identification and support the potential establishment of a GI, thereby enhancing product value and promoting sustainable aquaculture practices.

### 4.6. Relationship Between Body Size and Key Nutritional Components

Correlation and linear regression analyses using body weight revealed clear size-related patterns in several nutritional traits. In particular, fat content increased markedly with fish size, indicating greater lipid accumulation in larger fish and a corresponding rise in energy density as fish grow. Mineral levels generally increased with body size, supporting the higher ash/mineral content typically found in larger fish and implying improved mineral-related nutritional value (e.g., bone health and neuromuscular function for Ca and Mg). In addition, the increase in Fe with size may reflect greater hemoprotein content in muscle, which can enhance the fish’s contribution to dietary iron intake. DHA showed a positive, though more moderate, association with fish size, suggesting that fatty acid composition may be influenced by growth but also depends on other factors such as diet and rearing conditions. Overall, the trend analysis indicates that larger butter catfish may provide enhanced nutritional benefits, particularly in terms of fat-derived energy and mineral content; however, because the regression was based on three size levels, these relationships should be interpreted as indicative trends and would be strengthened by analysis using individual fish-level data.

### 4.7. Microbiological Quality

The size-related pattern in the microbiological quality of butter catfish flesh is consistent with previous findings indicating that smaller fish, due to their higher surface area-to-volume ratio, are more susceptible to rapid biochemical and microbial activity because of increased exposure to the surrounding environment [22]. In contrast, the lower surface area-to-volume ratio of larger fish may limit microbial colonization and slow degradation processes, contributing to reduced microbial loads.

According to the Codex Alimentarius [18] and Thai Food and Drug Administration standards [55], fishery products with total viable counts below 10^5^–10^6^ CFU/g are considered microbiologically acceptable for human consumption. Therefore, all samples in this study were well within the acceptable safety range. The acceptable microbial levels observed across all size groups align with the pH values remaining within the normal range for fresh fish muscle, suggesting that post-mortem biochemical changes and microbial proliferation were effectively controlled regardless of fish size. This concordance between pH stability and low TVC further supports the overall freshness and good microbiological quality of the cage-reared butter catfish.

Total coliform levels decreased with increasing fish size, being highest in small fish, lower in medium fish, and below detection in large fish. This decreasing trend with increasing fish size may also be linked to differences in degradation kinetics, as smaller fish tend to undergo faster metabolic and microbial changes due to higher relative exposure and accelerated post-mortem processes [22]. The low coliform counts observed across all samples are indicative of effective water quality management and good sanitation practices during aquaculture operations.

*Escherichia coli*, *Staphylococcus aureus*, and *Salmonella* spp. were not detected in any sample, supporting the microbiological safety of the cage-reared butter catfish. The absence of these pathogenic bacteria, together with acceptable pH and TVC values, indicates that neither size group experienced conditions favorable for rapid microbial spoilage or contamination. These findings emphasize the importance of effective water quality control, biosecurity measures, and hygiene management in cage aquaculture systems to ensure the safety and quality of freshwater fish products [56,57].

## 5. Conclusions

Butter catfish aquaculture in Thailand, particularly in emerging GI-targeted areas such as the Pak Phanang Basin, faces persistent challenges related to inconsistent product quality, limited baseline compositional benchmarks, and the absence of evidence-based specifications that can be used for routine quality control and GI documentation. In this context, the present study provides practical value by demonstrating that body size is a major source of biological variation that should be accounted for when defining product standards and market-grade specifications. The compositional and microbiological baseline generated here can help stakeholders (farmers, processors, and regulators) establish clearer acceptance criteria, strengthen quality assurance practices, and support the scientific justification required for GI dossiers. However, the current work represents a single harvest period and farming area and evaluates dorsal muscle only, and the study relied on fish collected from a commercial farm where routine water-quality parameters were managed by the farmer but not systematically recorded as numerical data, the findings should be interpreted as baseline evidence specific to the Pak Phanang Basin rather than as representative of Thailand or broader regions. For these reasons, future studies should validate these patterns across multiple sites and seasons, incorporate consumer-oriented sensory profiling and shelf-life assessment, and develop advanced traceability and authentication markers to reinforce GI certification and ensure long-term quality consistency of Pak Phanang Basin butter catfish.

## Figures and Tables

**Table 1:** Proximate composition, total energy, pH, and color values of dorsal muscle from cage-reared butter catfish of different body sizes.

Parameters	Large (3–5 Fish/kg)	Medium (6–10 Fish/kg)	Small (12–15 Fish/kg)	*p*-Value
Proximate composition				
Moisture (g/100 g)	68.33 ± 0.04 ^b^	71.07 ± 1.58 ^a^	72.66 ± 0.40 ^a^	0.004
Protein (g/100 g)	16.60 ± 0.08	16.58 ± 0.29	15.28 ± 1.10	0.101
Fat (g/100 g)	13.35 ± 0.20 ^a^	10.02 ± 0.64 ^b^	8.78 ± 0.03 ^c^	0.001
Ash (g/100g)	1.52 ± 0.05 ^a^	1.59 ± 0.12 ^a^	1.38 ± 0.02 ^b^	0.034
Carbohydrate (g/100 g)	1.24 ± 0.13	0.75 ± 0.10	1.90 ± 0.47	0.221
Total Energy (cal/g)	2203 ± 20 ^a^	1856 ± 84 ^b^	1695 ± 5 ^c^	0.000
pH	6.66 ± 0.25 ^b^	6.65 ± 0.11 ^b^	7.02 ± 0.18 ^a^	0.028
Color				
L*	57.42 ± 4.98 ^ab^	60.17 ± 4.42 ^a^	48.91 ± 2.10 ^b^	0.036
a*	−2.41 ± 0.57	−2.98 ± 0.48	−2.49 ± 0.68	0.475
b*	6.88 ± 3.23	8.72 ± 3.83	9.23 ± 1.20	0.693

Values are expressed as mean ± standard deviation (SD) from triplicate determinations. Different letters within the same row indicate significant differences (*p* < 0.05). Color indices: L* = lightness, a* = redness–greenness, and b* = yellowness–blueness.

**Table 2:** Mineral profile of dorsal muscle from cage-reared butter catfish of different body sizes.

Mineral (mg/100 g)	Large (3–5 Fish/kg)	Medium (6–10 Fish/kg)	Small (12–15 Fish/kg)	*p*-Value
Calcium (Ca)	1455 ± 78 ^a^	1311 ± 67 ^a^	1043 ± 22 ^b^	0.002
Magnesium (Mg)	1310 ± 50 ^a^	1118 ± 34 ^b^	1114 ± 4 ^b^	0.043
Iron (Fe)	18 ± 3 ^a^	12 ± 3 ^b^	7 ± 1 ^c^	0.007
Zinc (Zn)	27 ± 3	24 ± 1	28 ± 2	0.181
Manganese (Mn)	6 ± 1	5 ± 1	5 ± 0	0.168
Sodium (Na)	1446 ± 98	1657 ± 87	1497 ± 75	0.150
Potassium (K)	11,420 ± 731	10,986 ± 501	10,971 ± 277	0.584

Values are expressed as mean ± standard deviation (SD) from triplicate determinations. Different letters within the same row indicate significant differences (*p* < 0.05).

**Table 3:** Amino acid profile of dorsal muscle from cage-reared butter catfish of different body sizes.

Amino Acid (mg/g)	Type	Large (3–5 Fish/kg)	Medium (6–10 Fish/kg)	Small (12–15 Fish/kg)
Alanine	Non-essential	32.37 ± 0.01 ^a^	28.40 ± 0.27 ^b^	32.39 ± 0.06 ^a^
Arginine	Conditionally essential	31.15 ± 0.04 ^a^	28.09 ± 0.03 ^b^	30.17 ± 0.18 ^a^
Aspartic acid	Non-essential	56.81 ± 0.10 ^a^	48.74 ± 0.01 ^c^	53.34 ± 0.11 ^b^
Cysteine	Non-essential	1.58 ± 0.02 ^a^	1.05 ± 0.04 ^b^	1.10 ± 0.02 ^b^
Cystine	Non-essential	ND	ND	ND
Glutamic acid	Non-essential	86.59 ± 0.33 ^a^	69.85 ± 0.37 ^c^	75.68 ± 0.18 ^b^
Glycine	Non-essential	29.37 ± 0.05 ^b^	29.20 ± 0.06 ^b^	33.64 ± 0.25 ^a^
Histidine	Essential	10.63 ± 0.01 ^c^	12.45 ± 0.12 ^b^	13.54 ± 0.12 ^a^
Isoleucine	Essential	21.62 ± 0.21 ^a^	19.99 ± 0.04 ^b^	21.38 ± 0.04 ^a^
Leucine	Essential	50.14 ± 0.15 ^a^	41.46 ± 0.16 ^c^	45.19 ± 0.12 ^b^
Lysine	Essential	50.15 ± 0.07 ^a^	42.95 ± 0.04 ^c^	46.06 ± 0.09 ^b^
Methionine	Essential	14.07 ± 0.15 ^a^	8.24 ± 0.01 ^c^	9.13 ± 0.10 ^b^
Phenylalanine	Essential	20.96 ± 0.05 ^b^	20.67 ± 0.22 ^b^	22.20 ± 0.02 ^a^
Proline	Non-essential	15.10 ± 0.34 ^c^	16.14 ± 0.03 ^b^	17.87 ± 0.45 ^a^
Serine	Non-essential	19.90 ± 0.15 ^b^	18.67 ± 0.09 ^c^	20.27 ± 0.07 ^a^
Threonine	Essential	21.75 ± 0.18 ^b^	20.10 ± 0.02 ^c^	22.05 ± 0.01 ^a^
Tryptophan	Essential	ND	ND	ND
Tyrosine	Conditionally essential	12.58 ± 0.49 ^b^	12.74 ± 0.07 ^b^	13.13 ± 0.15 ^a^
Valine	Essential	27.71 ± 0.17 ^a^	23.58 ± 0.16 ^c^	25.45 ± 0.26 ^b^

Values are expressed as mean ± standard deviation (SD) from triplicate determinations. Different letters within the same row indicate significant differences (*p* < 0.05). ND = not detected.

**Table 4:** Fatty acid profile of dorsal muscle from cage-reared butter catfish of different body sizes.

Fatty Acid (g/100 g)	Symbol (C:D)	Type	Large (3–5 Fish/kg)	Medium (6–10 Fish/kg)	Small (12–15 Fish/kg)
Caprylic acid	C8:0	MCFA	ND	ND	ND
Nomanoic acid	C9:0	MCFA	ND	ND	ND
Capric acid	C10:0	MCFA	ND	ND	ND
Undecanoic acid	C11:0	MCFA	ND	ND	ND
Lauric acid	C12:0	MCFA	0.28 ± 0.00 ^b^	0.32 ± 0.00 ^a^	0.22 ± 0.00 ^c^
Tridecanoic acid	C13:0	MCFA	ND	ND	ND
Myristic acid	C14:0	LCFA	1.30 ± 0.00 ^b^	1.27 ± 0.00 ^c^	1.71 ± 0.00 ^a^
Tetradecenoic acid	C14:1; cis-9	LCFA	ND	0.03 ± 0.00	ND
Pentadecanoic acid	C15:0	OCFA	0.28 ± 0.00 ^b^	0.26 ± 0.00 ^c^	0.39 ± 0.00 ^a^
Pentadecenoic acid	C15:1; cis-10	MUFA	ND	ND	ND
Palmitic acid	C16:0	LCFA	21.84 ± 0.02 ^b^	21.13 ± 0.02 ^c^	25.96 ± 0.02 ^a^
Palmitoleic acid	C16:1	MUFA	2.48 ± 0.00 ^c^	2.84 ± 0.00 ^b^	3.18 ± 0.00 ^a^
Heptadecenoic acid	C17:1	MUFA	0.17 ± 0.00 ^b^	0.18 ± 0.00 ^a^	0.02 ± 0.00 ^c^
Stearic acid	C18:0	LCFA	6.63 ± 0.02 ^b^	5.07 ± 0.02 ^c^	7.04 ± 0.00 ^a^
Oleic acid	C18:1	MUFA	27.00 ± 0.02 ^b^	27.63 ± 0.05 ^a^	26.10 ± 0.05 ^c^
Linoleic acid	C18:2	PUFA	14.79 ± 0.01 ^b^	15.45 ± 0.01 ^a^	5.93 ± 0.00 ^c^
Methyl linolelaidate	C18:2; trans-9,12	PUFA	ND	ND	ND
γ-linolenic acid, ALA	C18:3; all-cis-6,9,12	PUFA	0.24 ± 0.00 ^a^	0.18 ± 0.00 ^b^	0.07 ± 0.00 ^c^
α-linolenic acid, ALA	C18:3; all-cis-9,12,15	PUFA	1.09 ± 0.00 ^b^	1.15 ± 0.00 ^a^	0.20 ± 0.00 ^c^
Arachidic acid	C20:0	LCFA	0.22 ± 0.00 ^c^	0.24 ± 0.00 ^b^	0.30 ± 0.00 ^a^
Ecoseonic acid	C20:1; cis-11	MUFA	0.94 ± 0.00 ^c^	1.05 ± 0.00 ^b^	1.39 ± 0.00 ^a^
Ecosadienoic acid	C20:2; all-cis-11,14	PUFA	0.93 ± 0.01 ^a^	0.67 ± 0.01 ^b^	0.33 ± 0.00 ^c^
Dihomo-γ-linolenic acid	C20:3; all-cis-8,11,14	PUFA	1.07 ± 0.00 ^a^	0.46 ± 0.00 ^b^	0.16 ± 0.00 ^c^
Ecosatrienoic acid	C20:3; all-cis-11,14,17	PUFA	ND	ND	ND
Eicosapentaenoic acid, EPA	C20:5; all-cis-5,8,11,14,17	PUFA	0.43 ± 0.00 ^a^	0.42 ± 0.00 ^b^	ND
Behenic acid	C22:0	LCFA	ND	0.15 ± 0.01	ND
Erucic acid	C22:1	MUFA	ND	0.12 ± 0.00 ^b^	0.24 ± 0.00 ^a^
Docosahexaenoic acid, DHA	C22:6	PUFA	2.20 ± 0.01 ^a^	2.00 ± 0.01 ^b^	0.28 ± 0.00 ^c^
Lignoceric acid	C24:0	VLCFA	ND	ND	ND
Selacholeic acid	C24:1	MUFA	ND	0.11 ± 0.00 ^b^	0.23 ± 0.00 ^a^
**Fatty acid group**					
MCFA			0.28	0.32	0.22
LCFA			29.99	27.89	35.01
OCFA			0.28	0.26	0.39
MUFA			30.59	31.81	30.92
PUFA			20.75	20.33	6.97
VLCFA			0	0	0
UFA/SFA ratio			1.7	1.8	1.1

Values are expressed as mean ± standard deviation (SD) from triplicate determinations. Different letters within the same row indicate significant differences (*p* < 0.05). ND = not detected. MCFA = Medium-chain fatty acid, LCFA = Long-chain fatty acid, OCFA = Odd-chain fatty acid, MUFA = Monounsaturated fatty acid, PUFA = Polyunsaturated fatty acid, VLCFA = Very long-chain fatty acid, UFA = Unsaturated fatty acid, SFA = Saturated fatty acid.

**Table 5:** Microbiological characteristics of dorsal muscle from cage-reared butter catfish of different body sizes.

Microorganisms	Large (3–5 Fish/kg)	Medium (6–10 Fish/kg)	Small (12–15 Fish/kg)	Standard/Remark
Total Viable Count (CFU/g)	1.5 × 10^4^	1.0 × 10^3^	1.3 × 10^3^	Acceptable level (<10^5^ CFU/g) [18]
Total Coliform (MPN/100 g)	11	7.2	<3	Indicative of good hygienic quality
*Escherichia coli* (MPN/100 g)	ND	ND	ND	Must be absent
*Staphylococus aureus* (CFU/g)	ND	ND	ND	Must be absent
*Salmonella* spp. (CFU/25 g)	ND	ND	ND	Must be absent

ND = not detected.

## Data Availability

The original contributions presented in this study are included in the article. Further inquiries can be directed to the corresponding author.
